# Low energy consumption phosphorescent organic light-emitting diodes using phenyl anthracenone derivatives as the host featuring bipolar and thermally activated delayed fluorescence†

**DOI:** 10.1039/c8ra10658d

**Published:** 2019-02-27

**Authors:** Zhonghua Ye, Zhitian Ling, Minyu Chen, Jiali Yang, Shuanglong Wang, Yanqiong Zheng, Bin Wei, Chong Li, Guo Chen, Ying Shi

**Affiliations:** School of Materials Science and Engineering, Shanghai University Shanghai 200072 P. R. China yshi@shu.edu.cn; Key Laboratory of Advanced Display and System Applications, Ministry of Education, Shanghai University 149 Yanchang Road Shanghai 200072 P. R. China chenguo@shu.edu.cn; Nanjing University of Technology No. 30 South Puzhu Road, Pukou District Nanjing 211816 P. R. China

## Abstract

A novel host material featuring the characteristics of bipolarity and thermally activated delayed fluorescence, 10-(4-(5,5-dimethylbenzofuro[3,2-*c*]acridin-13(5*H*)-yl)phenyl)-10-phenylanthracen-9(10*H*)-one (DphAn-5BzAc), has been designed and synthesized. By employing this material as the host of green emitter Ir(ppy)_2_acac, we have fabricated phosphorescent organic light-emitting diodes (PhOLEDs) with two hosting schemes, which are the single host system consisting of DhAn-5BzAc and the co-host system with 1,3-bis(carbazolyl)benzene (mCP). We found that the co-host based PhOLED achieved very low energy consumption values at high brightnesses, which were only 0.5, 5.9 and 94.0 mW m^−2^ at 100, 1000 and 10 000 cd m^−2^, respectively. The extremely low energy consumption for DhAn-based PhOLEDs were attributed to the excellent bipolar transport properties and thermally activated delayed fluorescence characteristics.

## Abstract 2

A bipolar host material 10-(4-(5,5-dimethylbenzofuro[3,2-*c*]acridin-13(5*H*)-yl)phenyl)-10-phenylanthracen-9(10*H*)-one (DphAn-5BzAc) with TADF properties, has been synthesized.

## Graphical abstract

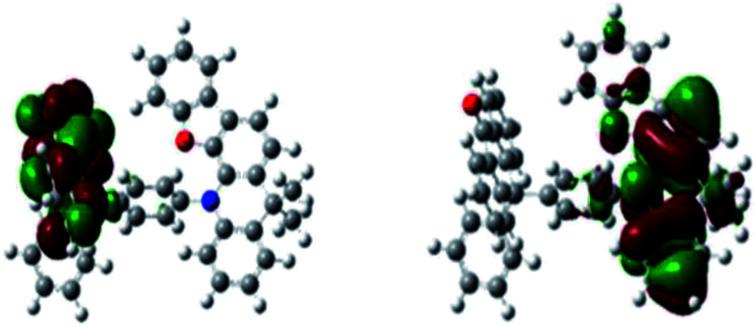

## Introduction

Organic light-emitting devices (OLEDs) have attracted intense interest due to the advantages of flexibility, high efficiency and eco-friendliness in the fields of full-color flat panel displays and solid-state lighting.^1–3^ Moreover, OLED displays also show considerable advantages over the tranditional displays in energy consumption. Over recent decades, low energy consumption OLEDs have developed rapidly under the driving force of commercial application requirements.

The low energy level barrier, balanced carrier transport, efficient host–guest system restrict the realization of low power consumption OLEDs,^4–6^ which are characterized with low turn-on voltage, high efficiency and importantly, reduced efficiency roll-off at high brightness. Recently, some efforts have been devoted to minimize the power consumption of OLEDs by employing highly efficient host materials and optimizing device structure. Commonly used hosts for phosphorescent emitters are conventional unipolar electron- or hole-transport materials, such as bis[2-(diphenylphosphino)phenyl]ether oxide (DPEPO), 1,3-bis(carbazolyl)benzene (mCP), 4,4′-bis(9-carbazolyl)-1,1′-biphenyl (CBP), which have been used to achieve high EQEs above 20%.^7–9^ However, due to the large energy gap between the singlet (S_1_) and triplet (T_1_) energies of those compounds, a high T_1_ is always accompanied with an even higher S_1_, leading to a mismatch in the frontier energy levels with the adjacent function layers, and consequently, a high triplet exciton density.^10,11^ This influence is detrimental to improve the efficiency of PhOLEDs, in virtue of the triplet–triplet annihilation (TTA) and triplet-polaron annihilation (TPA) processes. The use of a mixed host has been demonstrated as an effective way to improve the performance of PhOLEDs. In mixed host system, the balanced carrier transportation and minimization of carrier trapping by dopants by mixing two hosts are of great beneficial to PhOLEDs. For example, Lee *et al.*^12^ reported a green PhOLED with Ir(ppy)_3_ doped in a TCTA : TPBi mixed host, which reduced efficiency roll-offs by broadening the exaction recombination zone and reducing exaction leakage. Some combinations of hole-transporting and electron-transporting hosts result in the formation of exciplexes, which endow the devices with low driving voltages and low efficiency roll-off.^13–19^ Bipolar hosts integrating hole-transporting and electron-transporting units into one molecule have been extensively studied for highly efficient PhOLEDs.^20–23^ Recently, a potential approach is to use materials with thermally activated delayed fluorescence (TADF) as a host for improve the efficiency of OLEDs, by the utilization of TADF molecules as host material for PHOLED to enable full singlet and triplet energy transfer to emitter as well as decrease the device efficiency roll-off by virtue of efficient upconversion from triplet to singlet.^24–27^ Therefore, OLED performance can be dramatically improved by using a suitable host material and device structure. Simultaneously, power consumption can also be minimized due to the higher efficiency, lower driving voltage, and reduced efficiency roll-off at high brightness.

A rational molecular structure design strategy to obtain efficient bipolar TADF host materials was presented to combine an electron-donating and -withdrawing functionality in a twisted molecular framework. The acridine-based moieties, which possess excellent electron donating ability and high thermal stability, are qualified as the donors of efficient TADF materials.^23^ And the anthrone unit can be applied as acceptors to the bipolar molecules, due to their strong electrons withdrawing properties. Based on the above approaches, we designed and synthesized a new host compound (DphAn-5BzAc) for green PhOLEDs. The DphAn-5BzAc exhibits a small Δ*E*_ST_, affording an efficient reverse intersystem conversion (RISC). In addition, the host molecule shows excellent bipolar property, which is beneficial to balanced carrier transport in light-emitting layer. By doping a green emitter, bis(2-phenylpyridine)iridium(iii) acetylacetonate [Ir(ppy)_2_acac] in this TADF host, a high-efficiency green PhOLEDS with low turn-on voltage of 2.0 V was achieved. Furthermore, by mixing 1,3-bis(carbazolyl)benzene (mCP) and new materials as the host with better bipolar host material to ensure good carrier transport capability, the DphAn-5BzAc based co-host device revealed excellent performance and extremely low energy consumption with 0.5, 5.9 and 94 mW m^−2^ at 100, 1000 and 10 000 cd m^−2^, respectively.

## Experimental

### General information

All chemicals and reagents were used as received from commercial sources without further purification unless stated otherwise. The auxiliary materials for OLED fabrication such as dipyrazino[2,3-f:20,30-h] quinoxaline 2,3,6,7,10,11-hexacarbonitrile (HAT-CN), N0-diphenyl-[1,10-biphenyl]-4, 40-diamine (NPB), 1,3-bis(9-carbazolyl)ben-zene (mCP), bis(2-phenylpyridine)iridium(iii) acetylacetonate [Ir(ppy)_2_acac], 1,1-bis[4-(di-*p*-tolylamino)phenyl]cyclohexane (TAPC), 1,3,5-tris(*N*-phenylbenzimidazol-2-yl)benzene (TPBi), 1,3,5-tri(*m*-pyrid-3-yl-phenyl)benzene (TmPyPb) and lithium quinolinolate (Liq) were purchased from Lumtec Luminescence Technology Corp.

### Synthesis of materials

Synthesis of 10-(4-bromophenyl)-10-phenylanthracen-9(10*H*)-one (DphAn-Br): anthracene-9,10-dione (6.25 g, 30 mmol), ethylene glycol (1.99 g, 33 mmol), phosphoric acid (0.5 g), *o*-xylene (50 ml) were stirred in a three-necked flask under a nitrogen atmosphere. The solution was then refluxed for 14 h. After cooling to room temperature, the solution was extracted with ethyl acetate and distilled water. The organic layer was dried over anhydrous MgSO_4_ and evaporated using a rotary evaporator. The resulting residue was purified by column chromatography using petroleum ether/dichloromethane (6 : 1) to get the compound as a light-yellow powder intermediate-1 (3.86 g, 51%, HPLC = 98.7%).

Magnesium (0.24 g, 10 mmol) and 10 ml THF were stirred in a three-necked flask under a nitrogen atmosphere. After heated to 60 °C, 30 ml THF containing 1-bromo-4-iodobenzene (2.82 g, 10 mmol) was slowly dropwised. The solution was then refluxed for 4 h. After reaction, the mixture was cooled to room temperature and transfered to a constant pressure funnel. Intermediate-1 (2.52 g, 10 mmol) and THF (20 ml) were stirred in a three-necked flask under a nitrogen atmosphere, then the above mixture was slowly dropwised. After addition, the solution was then refluxed for 4 h. After cooling to room temperature, the solution was slowly poured into a dilute hydrochloric acid (8 wt%) with stirring for 40 min. The solution was then extracted with ethyl acetate and distilled water. The organic layer was dried over anhydrous MgSO_4_ and evaporated using a rotary evaporator. The resulting residue was purified by column chromatography using petroleum ether/toluene (5 : 1) to get the compound as a yellow powder. Above yellow power (4.2 g) and benzene (20 g) were stirred in a three-necked flask under a nitrogen atmosphere, boron trifluoride diethyl etherate (0.08 g, 0.5 mmol) was slowly dropwised. The solution was then heated to 65 °C and kept reaction for 8 h. After cooling to room temperature, the solution was extracted with ethyl acetate and distilled water. The organic layer was dried over anhydrous MgSO_4_ and evaporated using a rotary evaporator. The resulting residue was purified by column chromatography using petroleum ether/dichloromethane (5 : 1) to get the compound as a light-yellow powder intermediate-2 (1.31 g, 28%, HPLC = 98.5%).

Intermediate-2 (1.31 g, 2.8 mmol), toluene (30 ml) and HCOOH (5 ml) were stirred in a three-necked flask under a nitrogen atmosphere. The solution was then refluxed for 12 h. After cooling to room temperature, the solution was extracted with ethyl acetate and distilled water. The organic layer was dried over anhydrous MgSO_4_ and evaporated using a rotary evaporator. The resulting residue was purified by column chromatography using petroleum ether/dichloromethane (6 : 1) to get the compound as a white powder DphAn-Br (0.9 g, 75%, HPLC = 99.0%). ^1^H NMR (400 MHz, CDCl_3_): *δ* 8.22 (dd, 2H), 7.45–7.35 (m, 4H), 7.30–7.26 (m, 2H), 7.18–7.14 (m, 3H), 7.08–7.05 (m, 2H), 6.93–6.90 (m, 2H), 6.84–6.80 (m, 2H). ^13^C NMR (100 MHz, CDCl_3_): *δ* 184.48, 148.88, 145.88, 145.59, 132.91, 132.30, 131.97, 131.30, 130.58, 130.06, 128.33, 127.62, 127.57, 127.12, 121.17, 57.91. Elemental analysis (C_26_H_17_BrO): C, 73.07%; H, 4.02%; Br, 18.70%.

Synthesis of 10-(4-(5,5-dimethylbenzofuro[3,2-*c*]acridin-13(5*H*)-yl)phenyl)-10-phenylanthracen-9(10*H*)-one (DphAn-5BzAc) : 5,5-dimethyl-5,13-dihydrobenzofuro[3,2-*c*]acridine (2.99 g, 10 mmol), 10-(4-bromophenyl)-10-phenylanthracen-9(10*H*)-one (DpAn-Br) (4.68 g, 11 mmol), Pd_2_(dba)_3_ (0.1 g, 0.11 mmol) and potassium *t*-butoxide (3.37 g, 30 mmol) and toluene (100 mL) were stirred in a three-necked flask under a nitrogen atmosphere. The solution was then refluxed for 12 h. After cooling to room temperature, the solution was extracted with ethyl acetate and distilled water. The organic layer was dried over anhydrous MgSO_4_ and evaporated using a rotary evaporator. The resulting residue was purified by column chromatography using petroleum ether/dichloromethane (5 : 1) to get the compound as a yellow powder (5.27 g, 82%). ^1^H NMR (500 MHz, CDCl_3_) *δ* 8.34 (dd, *J* = 7.7, 1.5 Hz, 2H), 7.85 (d, *J* = 7.1 Hz, 1H), 7.59–7.43 (m, 7H), 7.39–7.32 (m, 3H), 7.31–7.27 (m, 4H), 7.15–7.08 (m, 5H), 7.05–6.99 (m, 2H), 6.94 (dd, *J* = 8.1, 0.9 Hz, 1H), 5.30 (s, 2H), 1.71 (s, 6H). ^13^C NMR (150 MHz, CDCl_3_) *δ* ppm 184.6, 155.6, 149.4, 146.4, 145.3, 144.4, 142.6, 140.8, 133.0, 132.7, 132.6, 132.4, 130.8, 130.7, 130.2, 128.1, 128.0, 127.5, 127.3, 127.1, 126.9, 126.5, 126.4, 125.0, 124.0, 123.8, 122.5, 122.1, 120.3, 119.8, 116.6, 113.4, 111.2, 58.1, 36.9, 31.0). Elemental analysis (C_47_H_33_NO_2_): C 87.04%, H 5.13%, N 2.45%.

### Measurements

^1^H NMR and ^13^C NMR spectra were recorded on a Bruker AV-600 spectrometer at room temperature. Elemental analysis was recorded on Elementar Vario MACRO cube. UV-vis absorption spectra were recorded on a UV-2501PC instrument. Photoluminescence spectra were taken using a FLSP920 fluorescence spectrophotometer, both in solution and in the solid state. Cyclic voltammetry was carried out using a CH Instrument 660E electrochemical analyzer and with a Ag/AgCl electrode as the reference electrode, with tetra(*n*-butyl) ammonium hexa-fluorophosphate (TBAPF6) in DMF as the supporting electrolytes. The glass transition temperatures (*T*_g_) of the compounds were determined under a nitrogen atmosphere using differential scanning calorimetry on a TA Q500 HiRes thermal analyzer with a scanning rate of 10 °C min^−1^ with nitrogen flushing. The decomposition temperature corresponding to 5% weight loss was conducted on a TA Q500 HiRes TGA thermal analyzer.

### Device fabrication and measurement

The devices were fabricated using conventional vacuum deposition of the organic layer and cathode onto an indiumtin-oxide (ITO) coated glass substrate under a base pressure lower than 5.0 × 10^−5^ mbar. Prepared glass substrates were cleaned using detergent, de-ionized water, acetone, and isopropanol. Immediately prior to loading into a custom-made high vacuum thermal evaporation chamber, the substrates were treated to a UV-ozone environment for 15 min. Then, organic layers and a metal cathode layer were evaporated successively by using shadow mask. The host complexes in the films were deposited on the glass substrates through thermal evaporation under a 5.0 × 10^−5^ mbar pressure to determine the PL spectrum and material characteristics. The entire organic layers and the Al cathode were deposited without exposure to the atmosphere. The deposition rates for the organic materials, and Al were typically 1.0 and 5.0 Å s^−1^, respectively. The current density–voltage–luminescence (*J*–*V*–*L*) characteristics were measured using FS-1000GA Test System. The luminance and spectra of each device were measured in the direction perpendicular to the substrate.

## Results and discussion

### Synthetic scheme

**Scheme 1:**
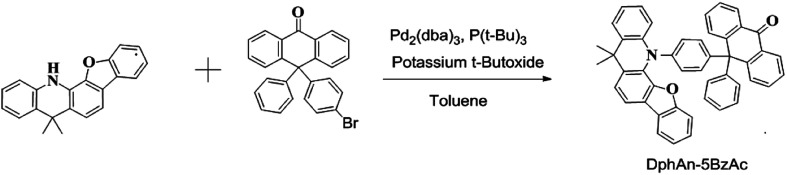
The synthetic route of DphAn-5BzAc.

Scheme 1 illustrates the molecular structures and the synthesis toward the host DphAn-5BzAc. The ancillary ligand 10-(4-bromophenyl)-10-phenylanthracen-9(10*H*)-one (DphAn-Br) was readily synthesized from 10*H*-spiro[anthracene-9,2′-[1,3]dioxolan]-10-one and 10,10-diphenyl-10*H*-spiro[anthracene-9,2′-[1,3]dioxolane] according with the ref. 23 (Scheme S1†). The target material DphAn-5BzAc was obtained by the Buchward–Hartwig couping reaction between corresponding intermediates and ancillary ligands in toluene. The details for the preparation of the compound are given in the Experimental section and ESI.†

### Theoretical calculations

**Fig. 1:**
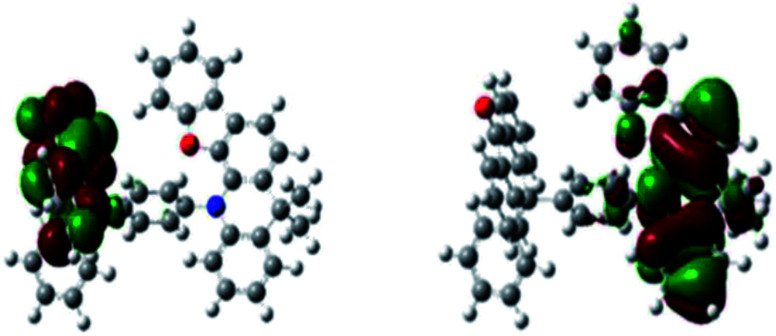
HOMO (left) and LUMO (right) of DphAn-5BzAc as computed in Gaussian 09 DFT B3LYP/6-31G* level of theory.

To gain further insight into the complex at the molecular level, the three-dimensional geometry and the frontier molecular orbital energy levels were calculated using density functional theory (DFT) at the B3LYP/6- 31G* level. A small Δ*E*_ST_ can be obtained by forcing the highest occupied molecular orbital (HOMO) and the lowest unoccupied molecular orbital (LUMO) to reside on different groups of the molecule. As shown in Fig. 1, the distribution of the HOMO is localized on DpAn unit, while the LUMO is localized on the acceptor unit of BzAc. The simulation Δ*E*_ST_ (S_1_–T_1_ gap) is 0.0017 eV, which was calculated by Gaussian 09 program in TDDFT B3LYP/6-31G* level of theory. The small Δ*E*_ST_ and significant separation of the HOMO and LUMO distribution can be observed, suggesting the possible existence of RISC process.

### Electrochemical properties

The electrochemical properties of synthesized compound were determined by cyclic voltammetry (CV) in THF solution. As shown in Fig. S1,† all of the compound present almost identical reduction in the CV curves, indicating formation of stable anion radical under the electrochemical condition, corresponding to the reduction process of electron accepter DpAn unit.

### Thermal properties

The thermal properties of the TADF material were investigated by thermal gravimetric analysis (TGA) and differential scanning calorimetry (DSC). As displayed in Table 1 and Fig. S2,† DphAn-5BzAc shows quite high decomposition temperatures (*T*_d_) of 370 °C. The glass transition (*T*_g_) of 118 °C was determined for DphAn-5BzAc from the DSC curve. The good thermal stabilities of this new TADF molecule, which attributed to the strong rigidity of these molecules, fulfil the requirement of film preparation *via* vacuum deposition.

**Table 1:** Summary of the physical properties of DphAn-5BzAc

Material	*T*_g_/*T*_m_/*T*_d_ [°C]	HOMO/LUMO^a^ [eV]	*E*_T_ b [eV]	Δ*E*_ST_^c^ [eV]	*λ*_abs_ d [nm]	*λ*_PL_ [nm]	*τ* e	*η*_PL_ e [%]
DphAn-5BzAc	118/249/370	−5.59/−2.52	2.97	0.05	335	512^e^/434^d^	22.51 ns/3.80 μs	76.2

a HOMO was determined using photoelectron spectroscopy. LUMO = HOMO + *E*_g_ where *E*_g_ is the optical band gap in film.

b Triplet energy corresponding to the first vibronic band of the phosphorescence spectra in frozen toluene (77 K).

c Δ*E*_ST_ = energy gap between S_1_ and T_1_.

d In toluene solution.

e Neat films were made on quartz substrates with a thickness of about 60 nm.

### Photophysical properties

**Fig. 2:**
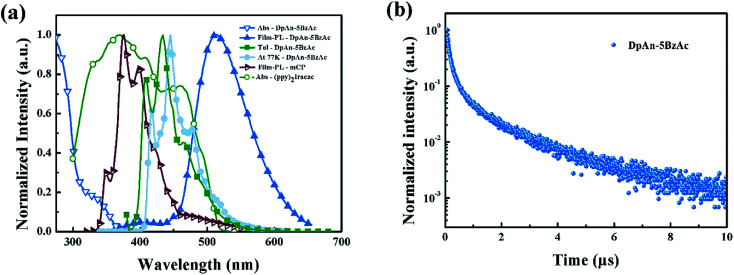
(a) UV-vis absorption (Abs) of Ir(ppy)_2_(acac) and absorption and PL spectra of mCP and DphAn-5BzAc in thin solid film at room temperature; (b) the transient decay curve of DphAn-5BzAc film at room temperature.

Fig. 2 describes the UV-vis absorption and PL spectra of DphAn-5BzAc at room temperature and 77 K, and the numerical data are summarized in Table 1. The absorption bands in the wavelength range of 280–350 nm for the compound exhibited n–π* and π–π* transitions. As shown in Fig. 2a, in toluene at room temperature, the maximum PL emission wavelengths of DphAn-5BzAc was observed at 434 nm. The big Stokes shift was observed, which inhibits the self-absorption of material. In toluene at 77 K, the phosphorescent spectra of the novel material show well defined vibronic structures, indicating that the lowest triplet state is LE state. The triplet energy of the host is found to be 2.97 eV, as determined from the highest energy peak of the spectra, which is sufficient to host green phosphorescent dopant. It is found that the Δ*E*_ST_ of the compound in toluene is calculated to be 0.05 eV. The small Δ*E*_ST_ of DphAn-5BzAc ensures a short TADF lifetime of 3.80 μs for its neat-film at room temperature (RT) along with a fluorescence lifetime of ns order (Fig. 2b). In addition, The PL quantum yields (PLQYs) of DphAn-5BzAc of neat-film is 76.2%, probably owing to the stronger rigidity.

### Carrier-transport properties

**Fig. 3:**
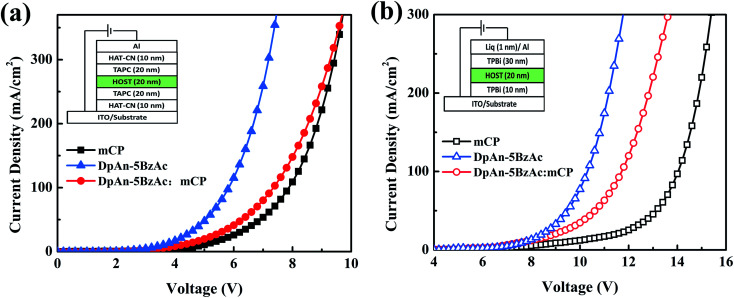
*J*–*V* characteristics of (a) hole-only devices and (b) electron-only devices for mCP, DphAn-5BzAc and DphAn-5BzAc : mCP.

In order to evaluate the carrier-transporting properties and find further support for the bipolar character of the title compound, hole-only (HOD) and electron-only devices (EOD) were fabricated. The construction of the hole-only device is ITO/HAT-CN (10 nm)/TAPC (20 nm)/DphAn-5BzAc or mCP (20 nm)/TAPC (20 nm)/HAT-CN (10 nm)/Al (100 nm), and the electron-only device is ITO/TPBi (30 nm)/DphAn-5BzAc or mCP (20 nm)/TPBi (30 nm)/Liq (1 nm)/Al (100 nm). The *J*–*V* plots (Fig. 3) show that the current densities of electron-only and hole-only devices based on DphAn-5BzAc are visibly higher than that of the host material mCP due to the excellent electron-donating ability of the donors and the good electron-withdrawing ability of the acceptor. And the slopes of the *J*–*V* curves of EOD and HOD based on DphAn-5BzAc are similar, which support the bipolar characteristics, resulting in the balanced carrier transport especially at the high voltage. Although the introducing of mCP has mitigated the *J*–*V* curve of the mixing-host cell due to the low carrier mobility, a superior bipolar property can be observed as a smaller difference between the *J*–*V* curves of hole- and electron-only devices based on the mixing the mCP and DphAn-5BzAc. The advanced bipolar may play an improved role in the carrier balance, which has positive influence in alleviating the roll-off of efficiency at high brightness.

### Electroluminescence properties

**Fig. 4:**
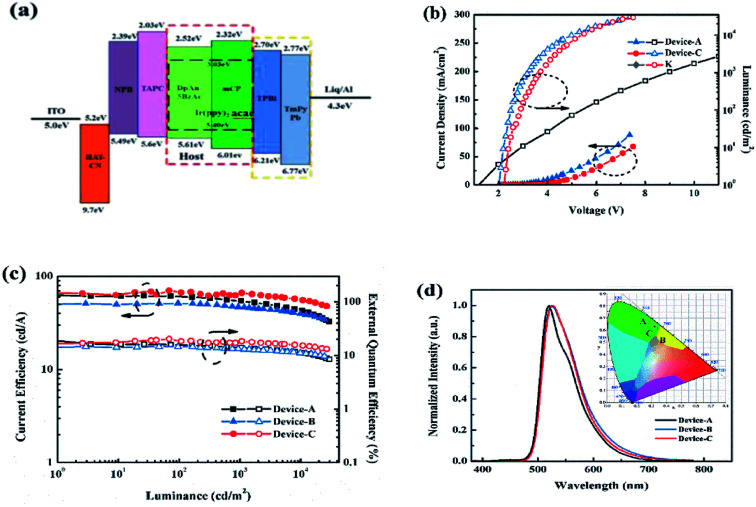
(a) Energy level diagram; (b) current density *versus* voltage *versus* luminance; (c) current efficiency *versus* luminance *versus* EQE; (d) EL spectra at 1000 cd m^−2^, inset: CIE coordinates of devices.

We fabricated the PhOLEDs using Ir(ppy)_2_acac, as green emitter in different matrix, mCP and DphAn-5BzAc for devices A and B, respectively. Fig. 4a displays the device configuration: indium tin oxide (ITO)/HAT-CN (10 nm)/NPB (50 nm)/TAPC (60 nm)/12 wt% Ir(ppy)_2_acac, host (40 nm)/TPBi : TmPyPb (1 : 1 35 nm)/Liq (1 nm)/Al (100 nm) is employed. In these devices, HAT-CN was employed as the hole injection layer (HIL), NPB and TAPC were used as the hole transport layers (HTLs). The mixing layer of TmPyPB and TPBi not only acts as an electron transporter, but also plays a significant role in blocking excess holes. To improve the EL efficiency of devices, we attempted to use a co-host system in EML, which is supposed to broaden the emission zone along with low probability of triplet–triplet exciton quenching.^28,29^ Hence, the mixed co-host of *N*,*N*′-dicarbazolyl-3,5-benzene (mCP) and DphAn-5BzAc with molar ratio of 1 : 1 was selected as an emitting layer (EML) for Ir(ppy)_2_acac, in device C. The device data obtained are shown in Fig. 4 and Table 2.

**Table 2:** Summary of the physical properties of DphAn-5BzAc

Devices	Von (V)	Maximum values			Values at 100 cd m^−2^	Values at 1000 cd m^−2^	Values at 5000 cd m^−2^	Values at 10 000 cd m^−2^
		CE (cd A^−1^)	EQE (%)	PE (lm/W)	V/CE/PC	V/CE/PC	V/CE/PC	V/CE/PC
A	3.1	63.1	19.37	63.9	4.1/60.7/0.72	5.1/54.2/9.54	6.3/47.2/61.3	7.0/43.1/165.6
B	2.0	51.2	14.5	66.9	2.5/51.3/0.5	3.2/46.7/6.7	4.2/42.3/49.1	5.0/39.5/125.9
C	2.3	70.0	20.3	85.8	2.9/67.3/0.5	3.7/66.4/5.9	4.7/59.4/39.1	5.4/55.6/94

As show in Fig. 4c, device B using DphAn-5BzAc as the host, is fairly stable with a maximum current efficiency, external quantum efficiency, and power efficiency of 51.2 cd A^−1^, 14.5%, and 66.9 lm W^−1^, respectively. Due to the proper energy levels of DphAn-5BzAc, device B reveals an extremely low turn-on voltage of 2.0 V. The more balanced carrier transport nature and TADF property of DphAn-5BzAc endow device B with a small efficiency roll-off at high luminance. Moreover, device B exhibits similar green emissions at 525 nm with the corresponding Commission Internationale de L'Eclairage (CIE) coordinates (0.34, 0.61), respectively, which are consistent with the PL spectra of Ir(ppy)_2_acac.^30^ As depicted in Table 2, the proper energy levels and the advanced carrier mobility of DphAn-5BzAc endow device B with an extremely low turn-on voltage. And for the comparative slight difference between the current efficiency, it is clear that the operating voltages of the devices differ greatly, so that the device B with a lower operating voltage has higher power efficiency and the reduced power consumption.

Given the large overlap spectra between the mCP and the Ir(ppy)_2_acac, which furnish the device A with a slightly higher performance, we introduced the mCP as the co-host. It is fascinating to mention that by using mCP and DphAn-5BzAc as an assistant co-host, the performance of device C, as summarized in Table 2, appears to be the best one among the single-host devices. The DphAn-5BzAc-based mix-host device C achieved a maximum current efficiency of 70 cd A^−1^, a maximum external quantum efficiency of 20.3% and a maximum power efficiency of 85.5 lm W^−1^. In addition, as shown in Fig. 4b, the turn-on voltage at 1 cd m^−2^ of device C is 2.3 V. Compared with device B, the increase in voltage, most likely, stemmed from the inferior charge transporting properties of EML due to the low mobility of mCP. The EL emission peak at 525 nm for device C, which shows that the device emits green light with the similar emission wavelength from Ir(ppy)_2_acac, due to the effective confinement of the triplet excitons within the EML.

One can find that the main difference between the two EML hosting schemes is the energy transfer from host(s) to guest. As for single-host devices, the carrier recombination occurs directly on Ir(ppy)_2_acac, due to the perfect energy level alignment of Ir(ppy)_2_acac, with adjacent layer (Fig. 4a).^31^ In contrast, the relevant excitons formation and transfer process for mixed host devices are found to be more complex. It can be understood from the energy level diagram shown in Fig. 4a that the excitons in the device C probably form partly in the host and partly in the dopant. The excitons in the host are then transferred into the dopant which then decays radiatively. For an efficient Förster energy transfer from the host to the dopant, there should be a strong overlap between the PL spectrum of the host and the absorption spectrum of the dopant.^32,33^ But no spectra overlap between the mCP and DphAn-5BzAc indicates the low possibility for efficient energy transfer. Thereby, the carrier recombination in EML of device C occurs on mCP and DphAn-5BzAc respectively and passes to the guest *via* the respective Förster transfer system. In consequence, on account of the introduction of mCP, the overlap between hosts and dopant is increased, and the energy transfer is correspondingly improved. The effective host-to-guest energy transfer endowed device C with a superior performance.

**Fig. 5:**
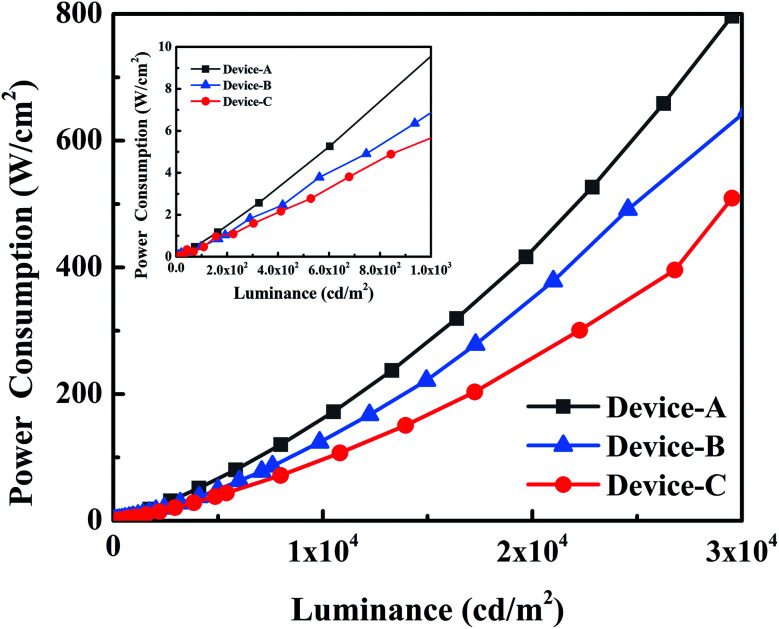
The power consumption *versus* luminance of devices.

It is worth noting that a series of devices with extremely low energy consumption are obtained based on the new host material, as shown in Fig. 5. Among these devices, the energy consumption of device C containing a hybrid EML was extremely low with 0.5, 5.9 and 94.0 mW m^−2^ at 100, 1000 and 10 000 cd m^−2^, respectively, which were lower than those of conventional LEDs. We attribute the superior performance of mCP : DphAn-5BzAc co-host devices to the superior carrier balance and a small Δ*E*_ST_ (0.05 eV). The bipolar transport characteristic of DpAn derivative has effectively reduced the operating voltage and improved the CE. Thus, the low voltage restricted the non-radiative energy of the device, thereby reducing power consumption. As for the bipolar host material, Zhou *et al.*^34^ designed and synthesized a series of carbazole/triazole (TAZ)-based bipolar materials. By using this bipolar materials as host, an Ir complex-based PhOLEDs achieved current efficiency of 28 cd A^−1^ at 1000 cd m^−2^. In comparison, we found that the PhOLEDs based on DphAn-5BzAc achieved CE of 39.3 cd A^−1^ at 1000 cd m^−2^, respectively, with corresponding excellent performances at the high practical brightness. On the other hand, the PhOLEDs based on DphAn-5BzAc : mCP mix-host achieved max power efficiency (85.8 lm W^−1^) at 2.56 V, respectively, with corresponding excellent performances at low drive-voltage. The TADF property of novel materials had positive effect on alleviating the triplet–triplet exciton quenching because of the lower density of triplet excitons. Therefore, the excellent performances for DphAn-5BzAc based PhOLEDs were attributed to the excellent bipolar transport properties and a small singlet-triplet energy gap afforded efficient reverse intersystem crossing, reducing the triplet density of the host for PhOLEDs.

## Conclusion

In conclusion, we reported a novel bipolar and TADF host molecules, DphAn-5BzAc by connecting DpAn with donor unit *via* the phenylene π-bridges. The compound DphAn-5BzAc exhibits superior excellent bipolar transport properties and a small Δ*E*_ST_ afforded efficient RISC, thus reducing the triplet density of the host for PhOLEDs. Furthermore, DphAn-5BzAc based co-host device revealed an extremely low turn-on voltage of 2.3 V and a slightly higher device performance with a maximum current efficiency, external quantum efficiency, and power efficiency of 70.0 cd A^−1^, 20.3%, and 85.8 lm W^−1^, due to the more balanced carrier transport and energy transfer properties in EML. Particularly, the DphAn-5BzAc based co-host device revealed extremely low energy consumption with 0.5 mW m^−2^ at 100 cd m^−2^, 5.9 mW m^−2^ at 1000 cd m^−2^ and 94 mW m^−2^ at 10 000 cd m^−2^, respectively, which were comparable than those of conventional LEDs. These make DpAn derivative promising host for high performance and low energy consumption OLED and lighting applications at high luminance.

## Conflicts of interest

There are no conflicts to declare.

## Supplementary Material

RA-009-C8RA10658D-s001

## References

[cit1] Miao Y. Q., Wang K. X., Zhao B., Gao L., Tao P., Liu X. G., Hao Y. Y., Wang H., Xu B. S., Zhu F. R. (2018). High-efficiency/CRI/color stability warm white organic light-emitting diodes by incorporating ultrathin phosphorescence layers in a blue fluorescence layer. Nanophotonics.

[cit2] Wang S. L., Yang J. L., Xu T., Dou D. H., Tang Z. Y., Gao Z. X., Chen M. Y., Guo K. P., Yu J. S., Plain J., Bachelot R., Zhang J. H., Wei B. (2019). Highly efficient and foldable top-emission organic light-emitting diodes based on Ag-nanoparticles modified graphite electrode. Org. Electron..

[cit3] Wang S. L., Qiao M. Y., Ye Z. H., Dou D. H., Chen M. Y., Peng Y., Shi Y., Yang X. Y., Cui L., Li J. Y., Li C. J., Wei B., Wong W. Y. (2018). Efficient Deep-blue Electrofluorescence with an External Quantum Efficiency Beyond 10%. iScience.

[cit4] Yook K. S., Lee J. Y. (2012). Organic materials for deep blue phosphorescent organic light-emitting diodes. Adv. Mater..

[cit5] Wang S., Zhao L., Zhang B. H., Ding J. Q., Xie Z. Y., Wang L. X., Wong W.-Y. (2018). High-Energy-Level Blue Phosphor for Solution-Processed White Organic Light-Emitting Diodes with Efficiency Comparable to Fluorescent Tubes. iScience.

[cit6] Yook K. S., Lee J. Y. (2016). Bipolar host materials for organic light emitting diodes. Chem. Rec..

[cit7] Li J., Nomura H., Miyazaki H., Adachi C. (2014). Highly efficient exciplex organic light-emitting diodes incorporating a heptazine derivative as an electron acceptor. Chem. Commun..

[cit8] Gao Z., Wang F., Guo K., Wang H., Wei B., Xu B. (2014). Carrier transfer and luminescence characteristics of concentration-dependent phosphorescent Ir(ppy)_3_ doped CBP film. Opt. Laser Technol..

[cit9] Wang Q., Oswald I. W., Yang X. L., Zhou G. J., Jia H. P., Qiao Q. Q., Hoshikawa-Halbert J., Gnade B. E. (2015). Managing charge and exciton transporting behavior in white organic light-emitting devices for high power efficiency and superior color stability. Adv. Electron. Mater..

[cit10] Yu D. H., Zhao F. C., Han C. M., Xu H., Li J., Zhang Z., Deng Z. P., Ma D. D., Yan P. F. (2012). Ternary ambipolar phosphine oxide hosts based on indirect linkage for highly efficient blue electrophosphorescence: towards high triplet energy, low driving voltage and stable efficiencies. Adv. Mater..

[cit11] Van Eersel H., Bobbert P. A., Janssen R. A. J., Coehoorn R. (2016). Effect of Förster-mediated triplet-polaron quenching and triplet-triplet annihilation on the efficiency roll-off of organic light-emitting diodes. J. Appl. Phys..

[cit12] Kim S. H., Jang J., Yook K. S., Lee J. Y. (2009). Stable efficiency roll-off in phosphorescent organic light-emitting diodes. Appl. Phys. Lett..

[cit13] Park Y. S., Lee S., Kim K. H., Kim S. Y., Lee J. H., Kim J. J. (2013). Exciplex-forming co-host for organic light-emitting diodes with ultimate efficiency. Adv. Funct. Mater..

[cit14] Kim S. Y., Jeong W. I., Mayr C., Park Y. S., Kim K. H., Lee J. H., Moon C. K., Brütting W., Kim J. J. (2013). Organic light-emitting diodes with 30% external quantum efficiency based on a horizontally oriented emitter. Adv. Funct. Mater..

[cit15] Kim K. H., Lee S., Moon C. K., Kim S. Y., Park Y. S., Lee J. H., Lee J. W., Huh J., You Y., Kim J. J. (2014). Phosphorescent dye-based supramolecules for high-efficiency organic light-emitting diodes. Nat. Commun..

[cit16] Shin H., Lee S., Kim K. H., Moon C. K., Yoo S. J., Lee J. H., Kim J. J. (2014). Blue phosphorescent organic light-emitting diodes using an exciplex forming co-host with the external quantum efficiency of theoretical limit. Adv. Mater..

[cit17] Lee J. H., Cheng S. H., Yoo S. J., Shin H., Chang J. H., Wu C. I., Wong K. T., Kim J. J. (2015). An exciplex forming host for highly efficient blue organic light emitting diodes with low driving voltage. Adv. Funct. Mater..

[cit18] Shih P. I., Shu C. F., Tung Y. L., Chi Y. (2006). Efficient white-light emitting diodes based on poly(N-Vinylcarbazole) doped with blue fluorescent and orange phosphorescent materials. Appl. Phys. Lett..

[cit19] Lee S., Kim K. H., Limbach D., Park Y. S., Kim J. J. (2013). Low roll-off and high efficiency orange organic light emitting diodes with controlled co-doping of green and red phosphorescent dopants in an exciplex forming co-host. Adv. Funct. Mater..

[cit20] Feng Y. S., Li P., Zhuang X. M., Ye K. Q., Peng T., Liu Y., Wang Y. (2015). A novel bipolar phosphorescent host for highly efficient deep-red OLEDs at a wide luminance range of 1000-10000 cd m^−2^. Chem. Commun..

[cit21] Zhang T., Liang Y. J., Cheng J. L., Li J. Y. (2013). A CBP derivative as bipolar host for performance enhancement in phosphorescent organic light-emitting diodes. J. Mater. Chem. C.

[cit22] Guo K. P., Wang H. D., Wang Z. X., Si C. F., Peng C. Y., Chen G., Zhang J. H., Wang G. F., Wei B. (2017). *et al.*, Stable green phosphorescence organic light-emitting diodes with low efficiency roll-off using a novel bipolar thermally activated delayed fluorescence material as host. Chem. Sci..

[cit23] Chen M. Y., Yang J. L., Ye Z. H., Wang S. L., Tang Z. Y., Chen G., Zheng Y. Q., Shi Y., Wei B., Wong W. Y. (2018). Extremely low efficiency roll-off of phosphorescent organic light-emitting diodes at high-brightness based on acridine heterocyclic derivatives. J. Mater. Chem. C.

[cit24] Peng T., Li G. M., Ye K. Q., Huang S., Wu Y., Liu Y., Wang Y. (2013). Concentration-insensitive and low-driving-voltage OLEDs with high efficiency and little efficiency roll-off using a bipolar phosphorescent emitter. Org. Electron..

[cit25] Lin C. C., Huang M. J., Chiu M. J., Huang M. P., Chang C. C., Liao C. Y., Chiang K. M., Shiau Y. J., Chou T. Y., Chu L. K., Lin H. W., Cheng C. H. (2017). Molecular design of highly efficient thermally activated delayed fluorescence hosts for blue phosphorescent and fluorescent organic light-emitting diodes. Chem. Mater..

[cit26] Liu X. Y., Liang F., Yuan Y., Cui L. S., Jiang Z. Q., Liao L. S. (2016). An effective host material with thermally activated delayed fluorescence formed by confined conjugation for red phosphorescent organic light-emitting diodes. Chem. Commun..

[cit27] Wang H., Meng L. Q., Shen X. X., Wei X. F., Zheng X. L., Lv X. P., Yi Y. P., Wang Y., Wang P. F. (2015). Highly efficient orange and red phosphorescent organic light-emitting diodes with low roll-off of efficiency using a novel thermally activated delayed fluorescence material as host. Adv. Mater..

[cit28] Chowdhury R., Ong T. S., Kee Y. Y., Yap S. S., Tou T. Y. (2015). Numerical and experimental studies of mixed-host organic light emitting diodes. Curr. Appl. Phys..

[cit29] Lee J. H., Wu C. I., Liu S. W., Huang C. A., Chang Y. (2005). Mixed host organic light-emitting devices with low driving voltage and long lifetime. Appl. Phys. Lett..

[cit30] Adachi C., Baldo M. A., Thompson M. E., Forrest S. R. (2001). Nearly 100% internal phosphorescence efficiency in an organic light-emitting device. J. Appl. Phys..

[cit31] Chatterjee T., Hung W. Y., Tang W. F., Chen H. F., Wong K. T. (2017). Carbazole-bridged triphenylamine-bipyridine bipolar hosts for high-efficiency low roll-off multi-color PhOLEDs. Org. Electron..

[cit32] Kozlov V. G., Bulovic V., Burrows P. E., Baldo M., Khalfin V. B., Parthasarathy G., Forrest S. R. (1998). Study of lasing action based on Förster energy transfer in optically pumped organic semiconductor thin films. J. Appl. Phys..

[cit33] Wei B., Kobayashi N., Ichikawa M., Koyama T., Taniguchi Y., Fukuda T. (2006). Organic solid laser pumped by an organic light-emitting diode. Opt. Express.

[cit34] Zhuang J. Y., Su W. M., Li W. F., Zhou Y. Y., Shen Q., Zhou M. (2012). Configuration effect of novel bipolar triazole/carbazole-based host materials on the performance of phosphorescent OLED devices. Org. Electron..

