# Supplementary material for: Low energy consumption phosphorescent organic light-emitting diodes using phenyl anthracenone derivatives as the host featuring bipolar and thermally activated delayed fluorescence
Source: RSC Adv. 2019 Feb 27;9(12):6881–9. doi: 10.1039/c8ra10658d (PMC9061084; doi:10.1039/c8ra10658d)
Supplement: RA-009-C8RA10658D-s001 [file RA-009-C8RA10658D-s001.pdf]

Electronic Supplementary Material (ESI) for RSC Advances.
This journal is © The Royal Society of Chemistry 2019

Low energy consumption phosphorescent organic light-emitting diodes using phenyl anthracenone derivatives as host featuring bipolar and thermally activated delayed fluorescence

Zhonghua Ye , Zhitian Ling , Minyu Chen , Jiali Yang , Shuanglong Wang , Yanqiong Zheng , Bin Wei , Chong Li, Guo Chen , Ying Shi

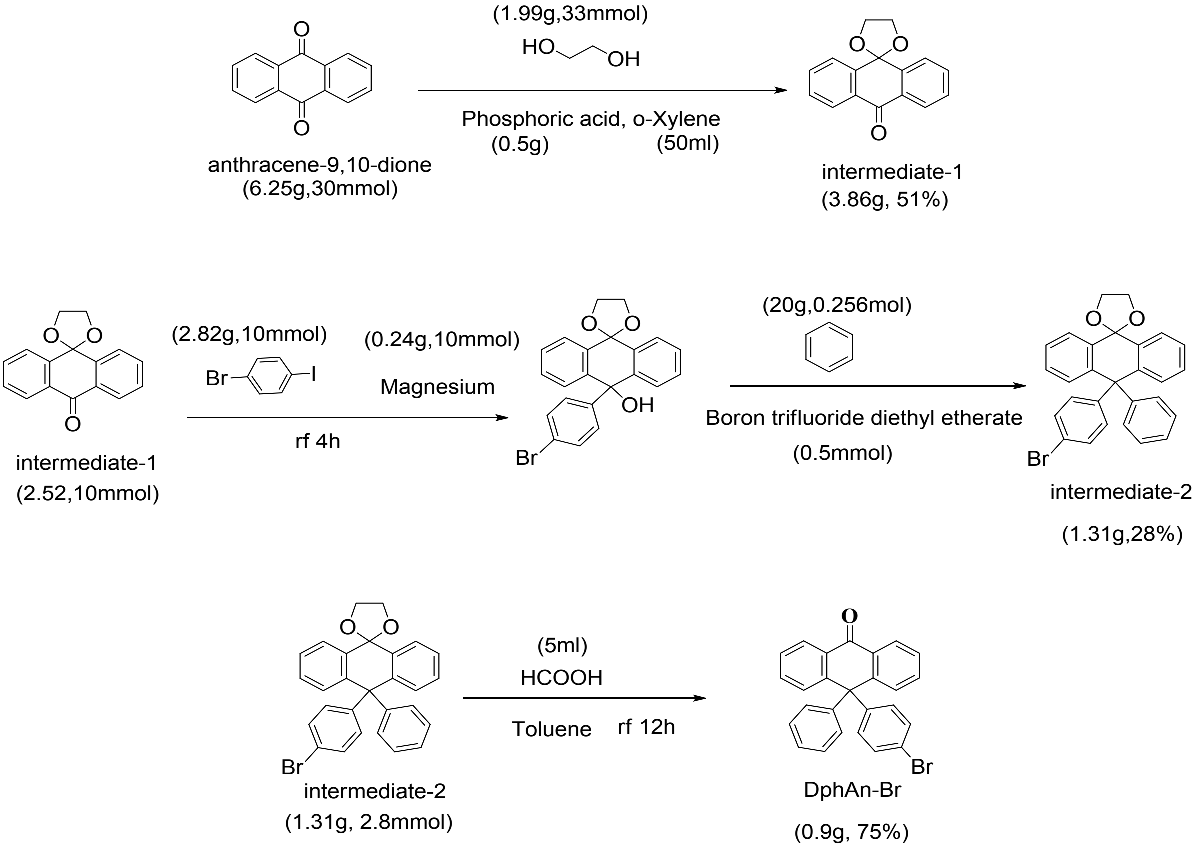

Scheme. S1 The synthetic route of DphAn-Br.

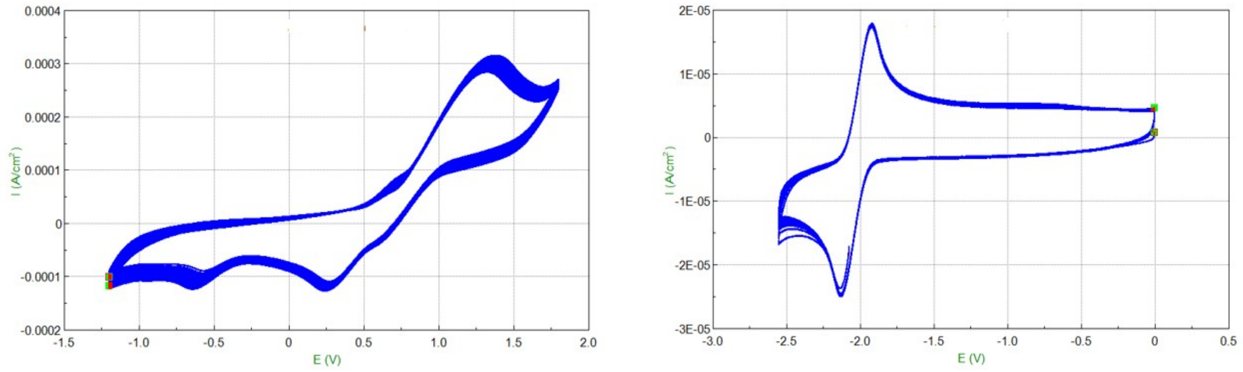

Fig. S1 Cyclic Voltammograms curves of DphAn-5BzAc: Oxidation potential (left) and Reduction potential(right).

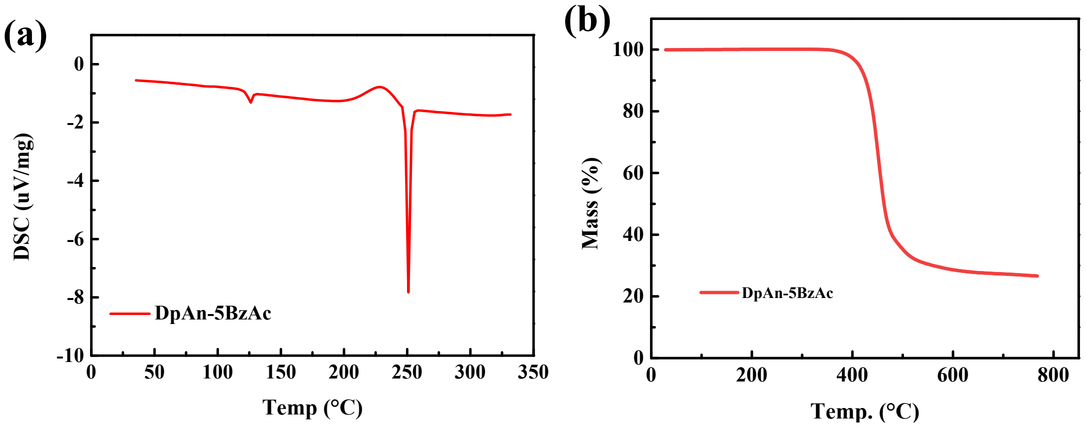

Fig. S2 Differential scanning calorimeter (DSC) curve and thermal gravimetric analysis (TGA) curve of DphAn-5BzAc.

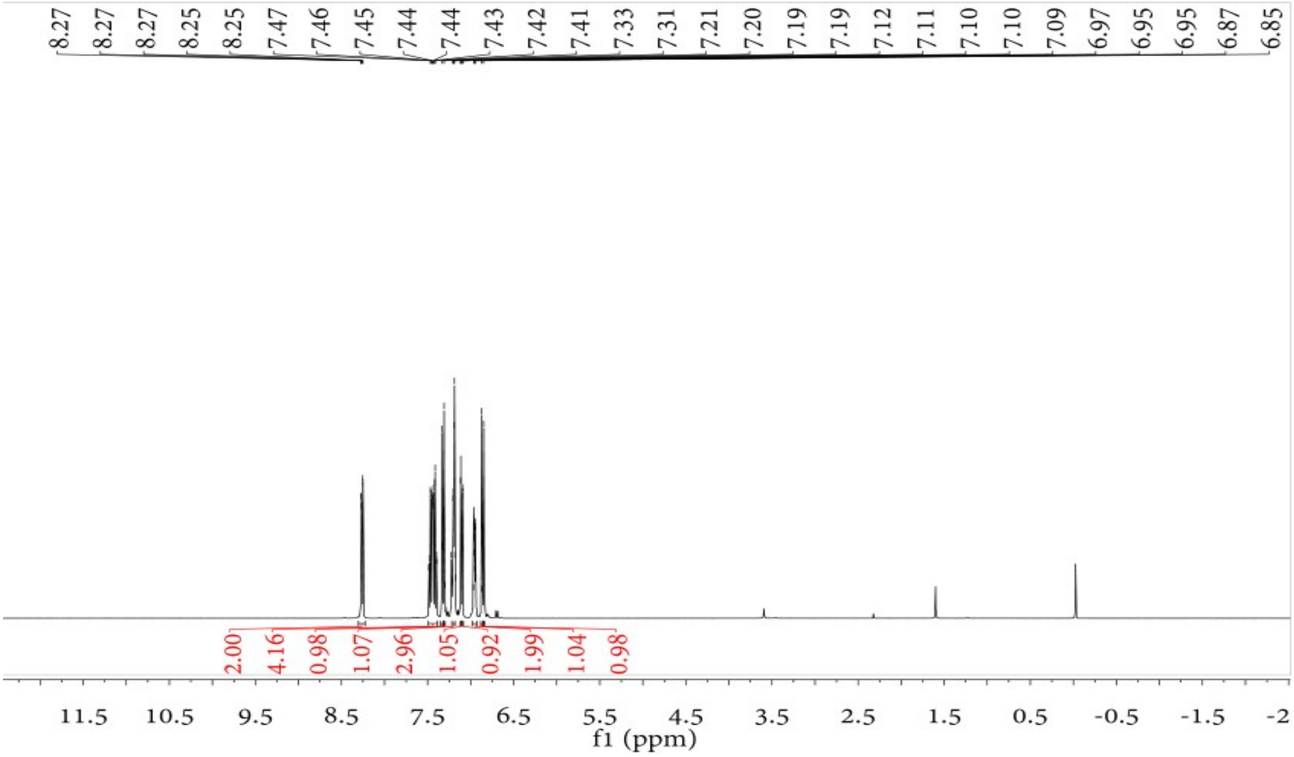

Fig. S3 The ^{1}H NMR spectra of DphAn-Br.

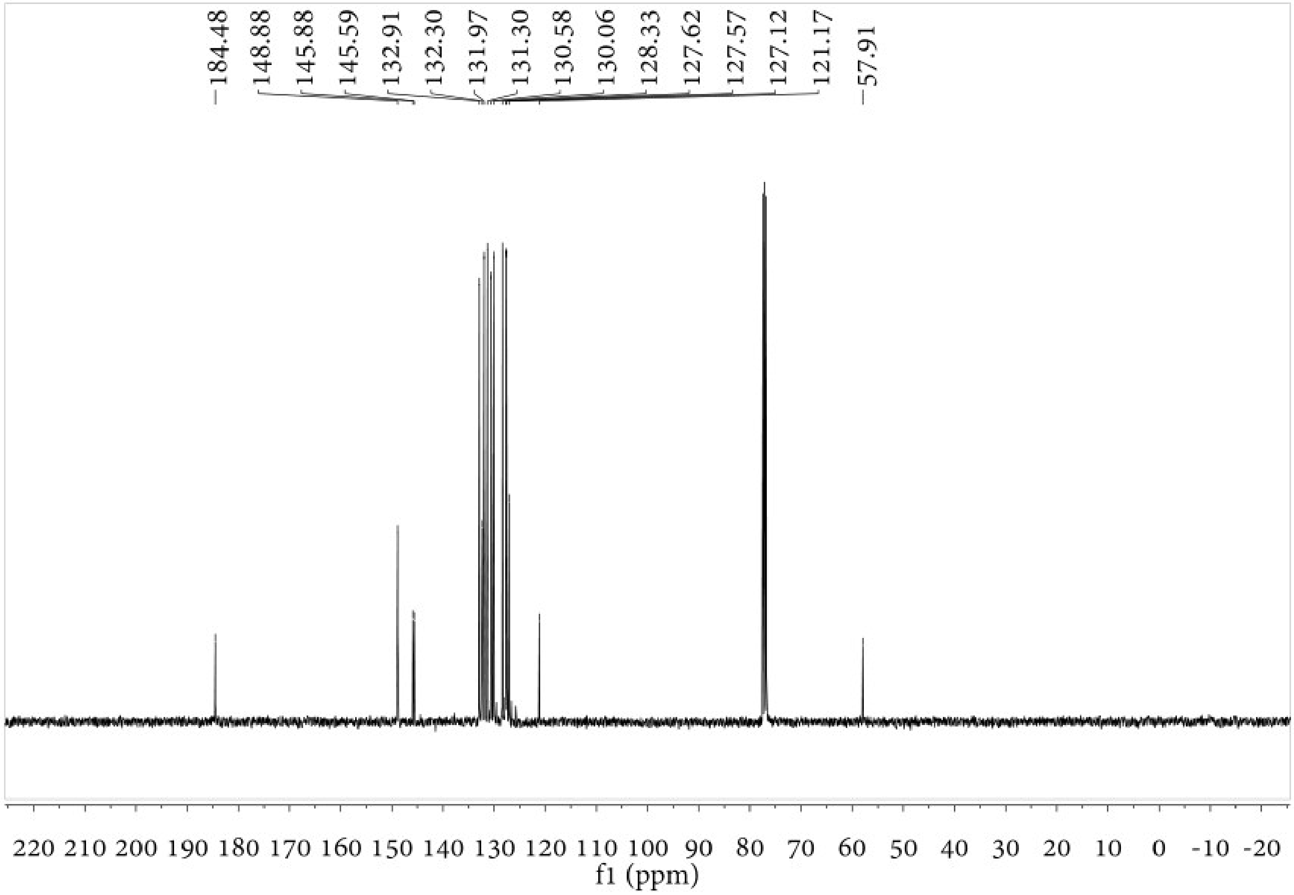

Fig. S4. The ^{13}C NMR spectra of DphAn-Br.

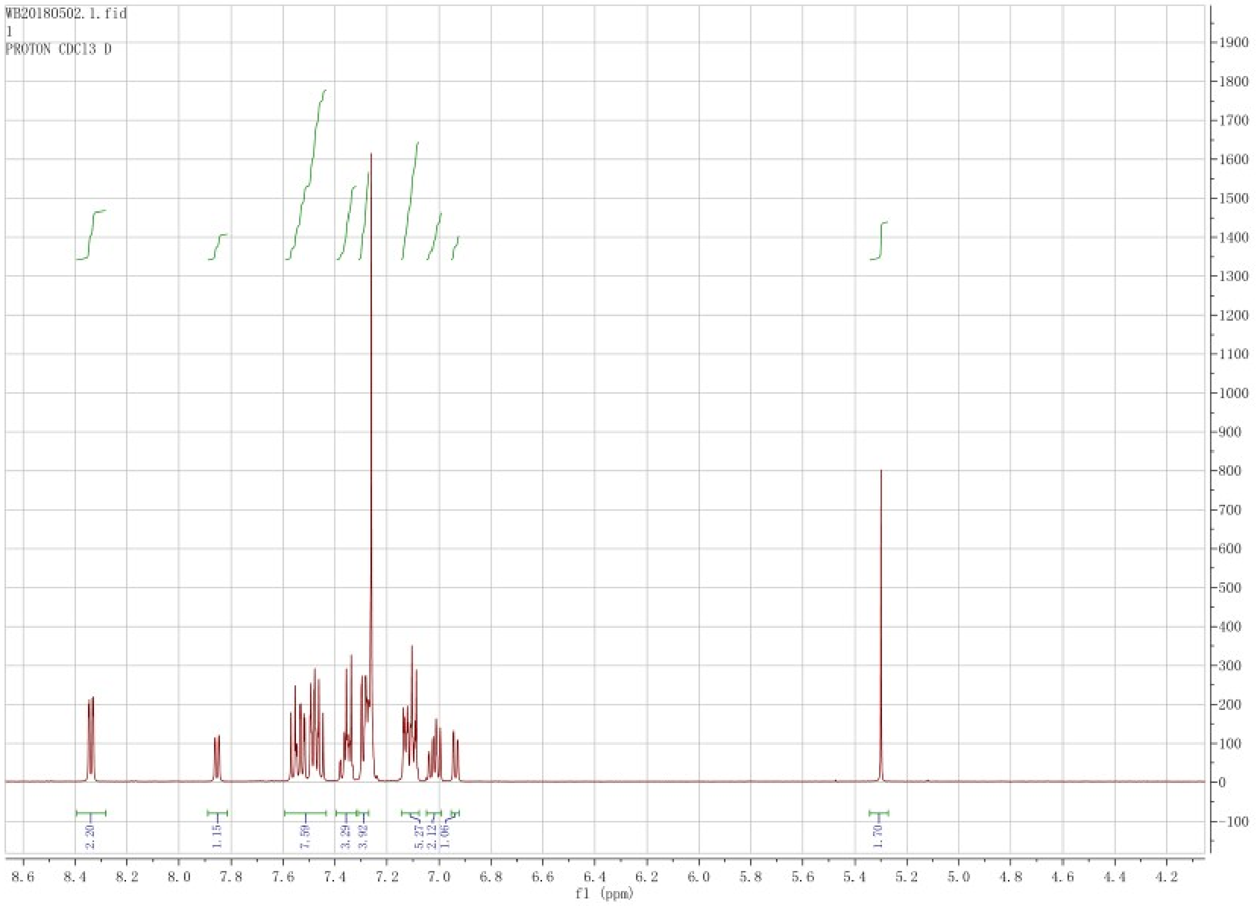

Fig. S5. The ^{1}H NMR spectra of DphAn-5BzAc.

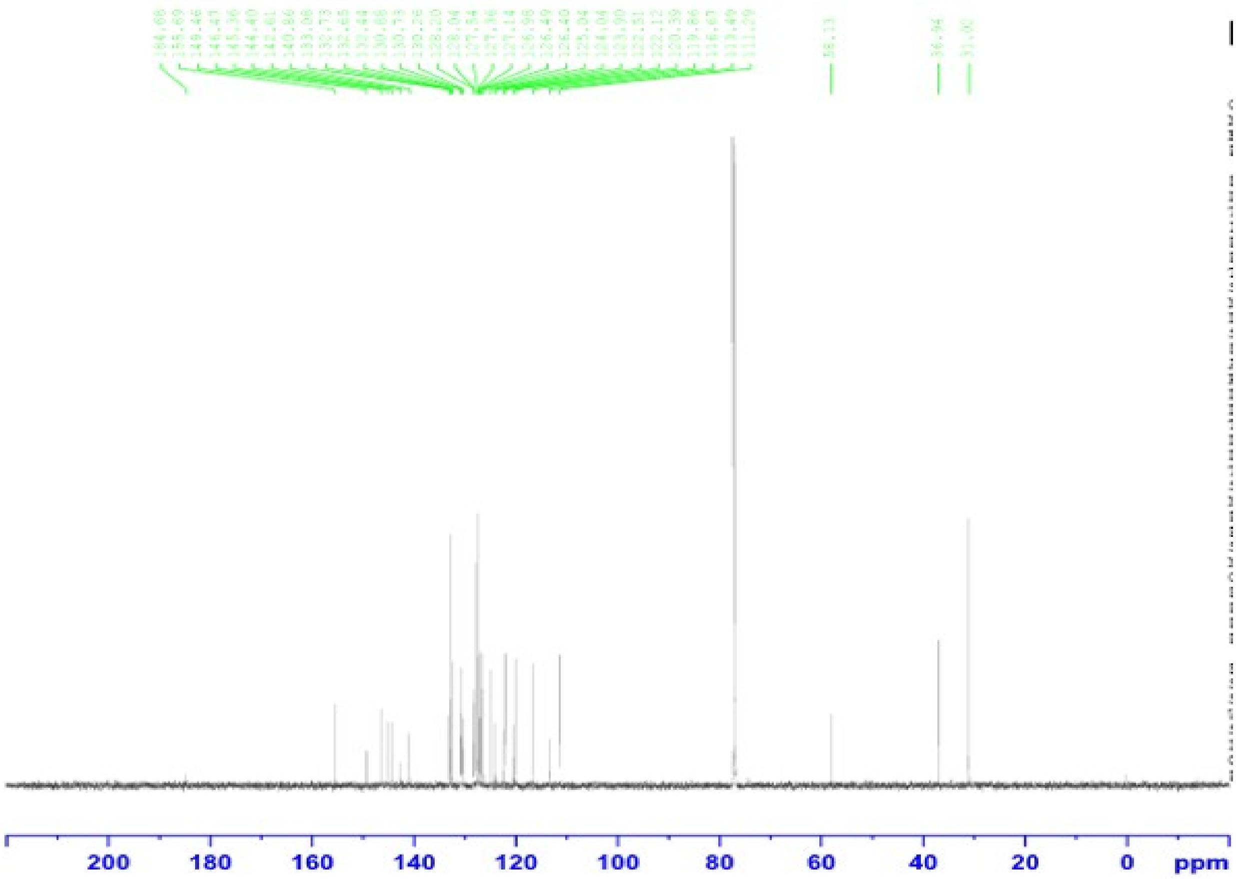

Fig. S6. The ^{13}C NMR spectra of DphAn-5BzAc.
